# The Great Masquerade: Not All Coronary Artery Stenosis Are Created Equal

**DOI:** 10.14797/mdcvj.1365

**Published:** 2024-05-20

**Authors:** Prasanti A. Kotta, Ajit K. Koduri, Jeffrey Berman, Veronica V. Rosen, Waleed T. Kayani

**Affiliations:** 1 Baylor College of Medicine, Houston, Texas, US

**Keywords:** vasospastic angina, intravascular ultrasound, intracoronary NTG, myocardial bridging

## Abstract

We present the case of a 60-year-old male, with active smoking and cocaine use disorder, who reported progressive chest pain. Various anatomical and functional cardiac imaging, performed to further evaluate chest pain etiology, revealed changing severity and distribution of left main artery (LMA) stenosis, raising suspicion for vasospasm. Intracoronary nitroglycerin relieved the vasospasm, with resolution of the LMA pseudostenosis. A diagnosis of vasospastic angina (VA) led to starting appropriate medical therapy with lifestyle modification counselling. This case highlights VA, a frequently underdiagnosed etiology of angina pectoris. We discuss when to suspect VA, its appropriate work-up, and management.

## Case Presentation

A 60-year-old male presented with progressive, intermittent episodes of chest pain. He described the pain as pressure-like, radiating to his left arm, present with both exertion and rest, and relieved by sublingual nitroglycerin (NTG). His past medical history was significant for atrial fibrillation, sick sinus syndrome with a dual-chamber pacemaker, a 30-pack/year smoking history, and cocaine use. Cardiovascular examination, including vital signs, were normal. Initial electrocardiogram (ECG) showed a paced rhythm without any abnormal ST-T changes. Serial ECGs and cardiac enzymes showed no dynamic changes. The urine drug screen was negative.

**Figure 1:**
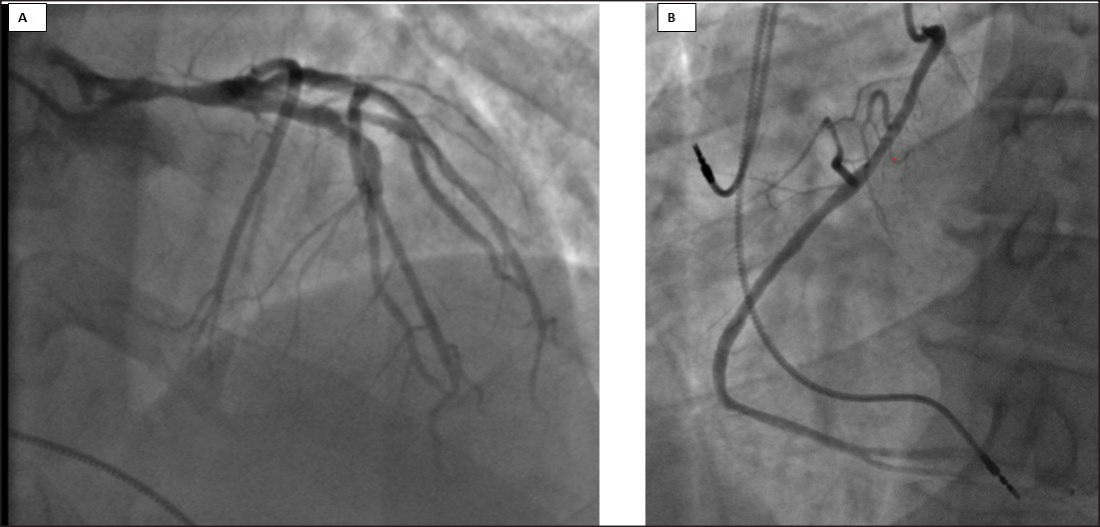
Initial coronary angiogram. **(A)** 80% stenosis of the left main artery, 40% stenosis in the proximal left anterior descending artery. **(B)** Right coronary artery had an anomalous origin from the ascending aorta.

Transthoracic echocardiography showed preserved ejection fraction without valvular or wall motion abnormalities. Myocardial perfusion with stress testing showed a moderate-sized territory of ischemia. Coronary angiography was subsequently performed and demonstrated 80% stenosis of the mid-left main artery (LMA) and 40% stenosis in the proximal-mid left anterior descending artery (LAD) (Figure 1). The right coronary artery (RCA) had an anomalous origin from the ascending aorta (Figure 1). No coronary intervention was performed, and cardiothoracic surgery was consulted for consideration of coronary artery bypass grafting given significant LMA stenosis. A coronary CT angiography (CCTA) to evaluate the anomalous course of the RCA was also recommended.

**Figure 2:**
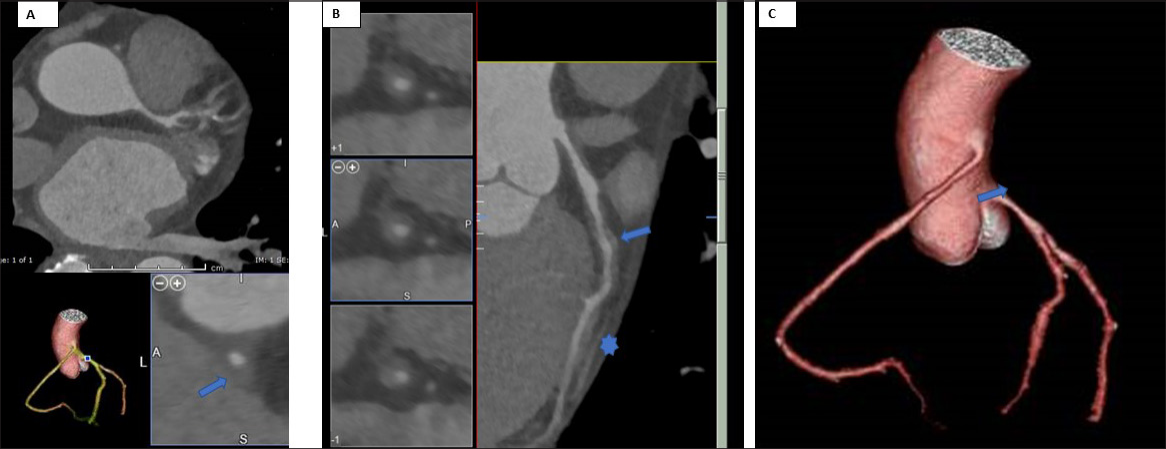
Coronary computed tomographic angiography (CCTA). **(A)** Diffuse narrowing of the left main artery with 70-80% maximal stenosis; CCTA reformat depicts diffuse narrowing of the left main artery resulting in 40-50% stenosis (arrow). **(B)** Curve planar reformat of the left anterior descending (LAD) shows diffuse circumferential wall thickening of the proximal LAD resulting in up to 50% stenosis (arrow). Incidental 1.4-cm myocardial bridge involves the mid-LAD without extrinsic compression or significant intraluminal stenosis (*). **(C)** 3-dimensional volume rendering of the coronary tree demonstrates high takeoff of the right coronary artery from the anterior surface of the ascending aorta (arrow).

CCTA showed severe diffuse narrowing of the LMA with 70% to 80% maximal stenosis, mild nonobstructive coronary artery disease (CAD) in the proximal LAD and mid-RCA (Figure 2). The RCA had a high, anterior take-off from the ascending aorta with the rest of the artery having a normal course. A 1.4-cm myocardial bridge involving the mid-LAD without significant stenosis was also identified. After a heart team discussion, the patient decided to pursue percutaneous coronary intervention.

**Figure 3:**
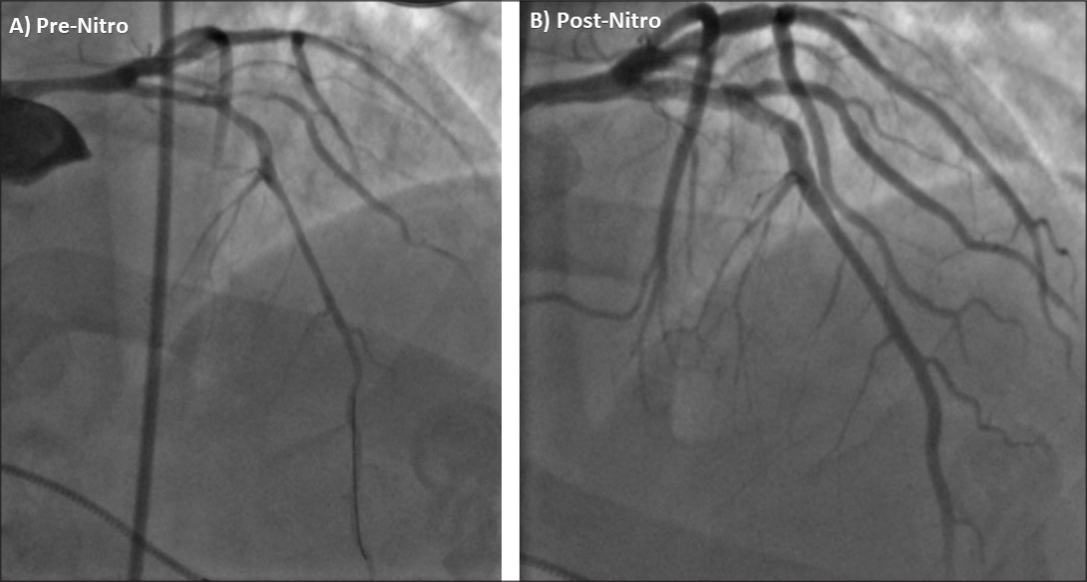
Repeat coronary angiogram. Pre-nitroglycerine coronary angiogram with changed severity and distribution of left main artery (LMA) stenosis compared to recent prior coronary angiogram (Figure 2). Coronary angiogram after intracoronary nitroglycerin administration with improvement in LMA stenosis and improved downstream blood flow.

**Figure 4:**
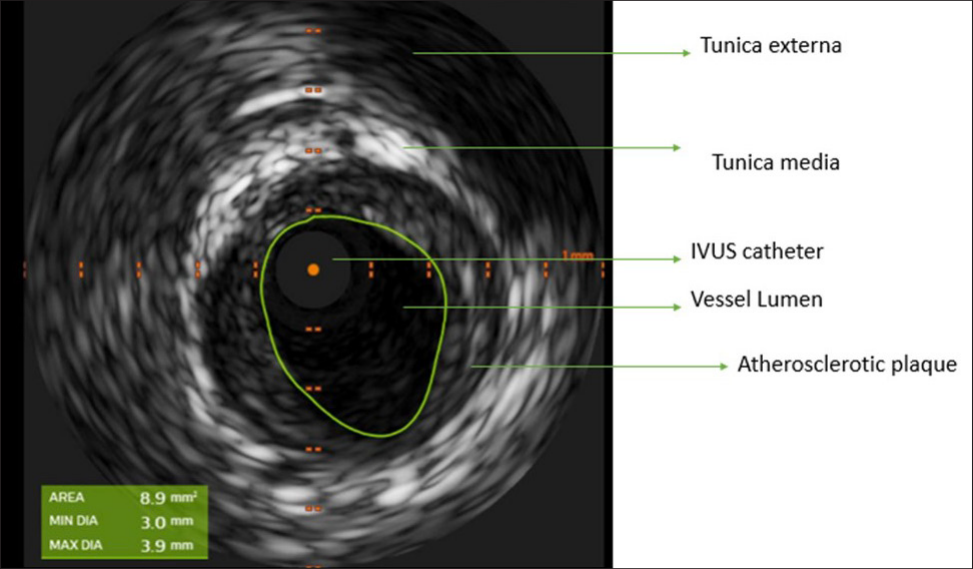
Intravascular ultrasound (IVUS) of the left main artery showing minimal luminal area of 8.9 mm^2^ and mild-moderate plaque burden.

Repeat coronary angiogram was performed (Figure 3A, Video 1). It revealed a significantly changed severity and distribution of LMA stenosis compared to the recent prior coronary angiogram, raising suspicion of coronary vasospasm. Intracoronary NTG was administered and relieved the vasospasm with improvement of LMA stenosis from 70% to 20% (Figure 3B, Video 2). Subsequently, intravascular ultrasound (IVUS) showed a minimal luminal area (MLA) of 8.9 mm^2^ with mild-moderate plaque burden in the LMA (Figure 4).

**Video 1:** Initial coronary angiogram pre-intracoronary nitroglycerin administration; see also at https://youtu.be/3oVymC12vP8.

**Video 2:** Repeat coronary angiogram after intracoronary nitroglycerin; see also at https://youtu.be/pXcMcIz8Vkw.

A diagnosis of vasospastic angina (VA) was made. The patient was started on diltiazem and counselled to avoid nonselective β blockers and common precipitating factors, including smoking, cocaine, and other recreational sympathomimetic agents. His diltiazem dose was increased in the following days, with the resolution of his chest pain. He was continued on aspirin and atorvastatin for management of coronary artery disease. At both the 3- and 6-month follow-ups, he remained free of chest pain.

## Discussion

This case highlights that VA can mimic atherosclerotic disease in its presentation and appearance on both anatomical and functional imaging. In our case, VA was present on the initial coronary angiogram, subsequent CCTA, and repeat coronary angiogram, masquerading as obstructive atherosclerotic disease. However, suspicion for VA was raised by the changed severity and distribution of LMA stenosis. Intracoronary NTG was used to test for resolution of vasospasm, with resolution of LMA stenosis. Secondly, IVUS was used to evaluate for plaque burden and other abnormalities, such as wall dissection and thrombus, and provided accurate evaluation of LMA lumen dimensions. Our case also highlights myocardial bridging (MB), a common anatomic variant associated with VA, and its implications on the management of VA with the coexistence of these two entities.

## Pathophysiology of VA

VA, also referred to as variant or Prinzmetal angina, is an uncommon type of angina caused by transient coronary vasospasm leading to downstream reduced myocardial blood supply. Although the spasm can occur in either a normal or a diseased coronary artery, it most commonly occurs within 1 cm of an atherosclerotic plaque.

The underlying pathophysiology for VA is multifactorial, with mechanisms including smooth muscle hyperreactivity, endothelial dysfunction, inflammation, altered autonomic responsiveness, and oxidative stress.^1^ The most well-documented risk factor is smoking. Common precipitants of VA episodes are stress, alkalosis, cold weather, cocaine, alcohol use, and triptans.^1,2,3^ Vasospasms frequently resolve spontaneously; however, persistent episodes can occur and lead to arrhythmia, syncope, myocardial infarction, and sudden death.^1,3^

## VA Diagnostic Criteria

VA usually does not occur with physical exertion or stress but rather during rest (typically at night or early morning), with transient ischemic changes on the electrocardiogram, and prompt resolution of angina either spontaneously or with nitrate administration. The diagnostic criteria for VA are summarized in Table 1.^2,4^

**Table 1:** Vasospastic angina diagnostic criteria.^1,2^


SPONTANEOUS EPISODE	PROVOKED EPISODE

Nitrate-responsive angina + transient ischemic electrocardiographic (ECG) changes **or** angiography documented coronary artery spasm (defined as > 90% coronary artery occlusion spontaneously)	Nitrate-responsive angina + transient ischemic ECG changes + angiography documented coronary artery spasm (defined as > 90% coronary artery occlusion with provocation)


## Diagnostic Studies

### Coronary Angiography and Provocative Testing

Coronary angiography should assess for the presence of obstructive atherosclerotic CAD and other forms of structural CAD (eg, coronary artery dissection). The diagnosis of VA is confirmed through coronary angiography by reversibility of the stenosis either through resolution of stenosis spontaneously or with NTG; through induction of stenosis by provocative testing with intracoronary agents such as ergonovine, acetylcholine, or methylergonovine; or through nonpharmacological means such as hyperventilation or cold pressor testing.^2,4^ A positive provocative test is defined in Table 1.^1,2,3,4^

There are no specific guidelines on when to initiate provocative testing or administer NTG.^1,2,4^ Therefore, a high index of suspicion is warranted to diagnose VA. Typical features that should raise suspicion for VA are reversibility of lesions, demonstrated on sequential ECG monitoring or significant changes in lesion size or severity on repeat coronary catheterization, IVUS findings that are inconsistent with a fixed, obstructive lesion, reversibility of the lesion on intracatheterization nitroglycerin, and positive provocative testing. It is important to remember that the presence of VA does not exclude underlying significant obstructive lesions; however, failure to correctly diagnose vasospastic disease can lead to incorrect treatment and unwarranted interventions, including percutaneous coronary intervention or coronary artery bypass grafting.

### Intravascular Ultrasound

IVUS can be used to rule out significant atherosclerotic plaque leading to stenosis and confirm the diagnosis of VA. Left main lesions with an MLA ≤ 5.9 mm^2^ on IVUS have been found to be reliable predictors of significant functional obstructive coronary artery disease in the LM, and deferral of intervention for an MLA ≥ 6 mm^2^ has been found to be safe and associated with favorable outcomes.^5,6^

### Coronary Computed Tomographic Angiography

Its utility in diagnosis of VA is limited by the transient nature of vasospasm. Furthermore, the use of nitroglycerin, which is commonly used to optimize CCTA image acquisition, may mask the diagnosis of VA by concurrently treating vasospasm. Prior studies have suggested protocols of CCTA pre- and post-nitrate therapy to detect VA, but limitations in clinical settings include a requirement for multiple CCTAs and a high index of suspicion for VA.^7^

## Management of VA

The key tenants of VA treatment are summarized in Table 2.^2,4^ The most important lifestyle modification is avoidance of triggering substances.^1,3,4,8^ Smoking is the most consistently demonstrated risk factor. Other precipitants include sympathomimetics, beta-blockers (through unopposed alpha constriction), stress, and cold, among other identified triggers.

**Table 2:** Vasospastic angina therapies.^1,2^


Avoid precipitants	Smoking Sympathomimetics – cocaine, methamphetamine, ecstasy, adrenaline Other agents – beta-blockers, ergot alkaloids Mental stress

Calcium channel blockers (first-line therapy)	Non-dihydropyridine (verapamil, diltiazem), dihydropyridine (nifedipine, amlodipine)

Antianginal therapy	Nitrates, nicorandil, statins, cilostazol

Refractory angina	Stellate ganglion block


The first-line treatment is calcium channel blockers (CCBs), considered to be cardioprotective as they have been shown to be an independent determinant of infarct-free survival. These include both the non-dihydropyridine (verapamil, diltiazem) and dihydropyridine (nifedipine, amlodipine) calcium channel blockers.

Second-line therapy includes other antianginal therapies such as long-acting nitrates, nicorandil, statins, and cilostazol. Nitrate tachyphylaxis limit their long-term utility, and several studies (mostly observational) have reported long-term use of nitrates to be associated with a higher incidence of cardiovascular events in patients with VA. Statin therapy, with antioxidant properties, can also improve endothelial function at sites of vasospasm and reduce recurrence.^1,3,8^ In addition, aerobic exercise training has also been shown to reduce angina episodes in patients with VA.

In patients who are unresponsive to maximal antianginal therapies, antinociceptive approaches such as stellate ganglion block may be required.^2,4^ If coexistent CAD is present, optimal management of CAD with goal-directed medical therapy is also pertinent.

## Long-term Management and Outcomes in VA

Therapies such as CCBs are effective in relieving coronary spasms and preventing clinical events. Therefore, the prognosis of VA is generally better than for significant atherosclerotic stenosis. However, coronary vasospasm is sometimes associated with fatal complications such as sudden death, ventricular arrhythmia, and myocardial infarction.^1,3^

Certain clinical factors have been associated with poor prognosis in patients with VA.^9^ These are summarized in Table 3.

**Table 3:** Prognostic factors in vasospastic angina.


Poor prognostic factors in vasospastic angina	Serious clinical presentations (ST-elevation, acute coronary syndrome, cardiac arrest), combined atherosclerotic stenosis, multi-vessel spasm, not using calcium-channel blockers, definite spasm on provocative testing, focal spasm


Many of these variables that determine a patient’s long-term prognosis can be obtained through invasive coronary angiography and provocation tests. Therefore, invasive angiography and provocation tests can be used to guide long-term VA management. In addition, continuing antiatherosclerotic treatments for accompanying atherosclerotic stenosis and maintaining CCB intake are important for improving the long-term prognosis of patients with VA. Furthermore, precipitating substances such as smoking should be avoided.

## Association with Myocardial Bridge

Our case also highlights myocardial bridging, a common anatomic variant associated with VA, and implications on the management of VA with the coexistence of these two entities.^10^ Myocardial bridging is a congenital, anatomic abnormality when a coronary artery, normally located in the epicardial layer, dives into the underlying myocardium. The intramural segment can collapse during systole. Although thought to be benign in most cases, the presence of myocardial bridging is associated with a higher risk of atherosclerosis and coronary vasospasm.^11^ The exact etiology of the latter association is uncertain but may be related to underlying vascular dysfunction at the sites of the coronary artery adjacent to the myocardial bridge. Nitrates have a relative contraindication in patients with myocardial bridging as they are thought to exacerbate compression of the myocardial bridge, thus in our case, nitrate therapy was not initiated.^11^

## Conclusion

Vasospasm can mimic atherosclerotic disease in its presentation, but a careful evaluation of anatomical and functional imaging can help distinguish the two. Characteristics such as dynamic changes in disease distribution on serial tests should raise suspicion for VA. A high index of suspicion is warranted to promptly use additional tools including IVUS, provocative testing, and NTG to diagnose VA. IVUS can characterize area of stenosis and help distinguish VA from atherosclerotic disease. Further work is still needed to understand the optimal diagnostic approach to evaluate vasospasm on noninvasive imaging modalities and coronary angiography. Failure to correctly diagnose VA can lead to incorrect treatment and unwarranted interventions. Correct management of VA includes avoidance of precipitating factors that potentiate coronary vasoconstriction. The main pharmacological therapy is calcium channel blockers.
